# Hypoxia induces H19 expression through direct and indirect Hif-1α activity, promoting oncogenic effects in glioblastoma

**DOI:** 10.1038/srep45029

**Published:** 2017-03-22

**Authors:** Weining Wu, Qi Hu, Er Nie, Tianfu Yu, Youzhi Wu, Tongle Zhi, Kuan Jiang, Feng Shen, Yingyi Wang, Junxia Zhang, Yongping You

**Affiliations:** 1 Department of Neurosurgery, The First Affiliated Hospital of Nanjing Medical University, Nanjing, Jiangsu 210000, China

## Abstract

H19 expression is elevated in many human tumors including glioblastomas, suggesting an oncogenic role for the long noncoding RNA; yet the upregulation of H19 in glioblastomas remains unclear. Here we report that hypoxia significantly stimulated H19 expression in glioblastoma cell lines, which was related to hypoxia-inducible factors 1α (Hif-1α). Hif-1α promoted H19 expression in U87 and U251 cells. Meanwhile PTEN is an advantageous factor to affect H19 expression, through attenuating Hif-1α stability. Hif-1α also positively correlates with H19 in human glioblastoma samples depending on PTEN status. ChIP and luciferase reporter assays showed that Hif-1α induced H19 transcription through directly binding to the H19 promoter. Furthermore, Hif-1α upregulated specific protein 1 (SP1) expression in glioblastomas cells *in vitro* and *in vivo*, and SP1 also strongly interacted with the H19 promoter to promote H19 expression under hypoxia. We also showed that H19 acts as a molecular sponge that binds miR-181d, relieving inhibition of β-catenin expression. Therefore, H19 participates in hypoxia-driven migration and invasion in glioblastoma cells. In summary, our results uncover the mechanisms that stimulate H19 expression under hypoxia to promote malignant effects in glioblastomas and suggest H19 might be a promising therapeutic target.

A hypoxic microenvironment is a common feature of solid tumors that is closely correlated to tumor progression and negatively influences overall prognosis^1^. Hypoxic states occur in tumors, including glioblastomas, when uncontrolled cell proliferation outpaces the available oxygen supply, leading to intratumoral necrosis^2^,^3^. Tumor hypoxia is associated with metastases^4^, recurrences^5^ and resistance to chemotherapy and radiation therapy^6^,^7^,^8^. These effects are mediated by a family of transcription factors called hypoxia-inducible factors 1α and 2α (Hif-1α and HIF-2α)^9^. Hif-1α expression and activity is highly dependent on oxygen supply; under normoxic conditions, Hif-1α is rapidly hydroxylated by prolyl hydroxylase domain (PHD)-containing proteins and subsequently polyubiquitinated by the von Hippel–Lindau tumor suppressor (pVHL) for degradation through the proteasome pathway^9^. Under hypoxic conditions, pVHL-mediated Hif-1α degradation is abolished, causing Hif-1α to accumulate and translocate to the nucleus where it forms a heterodimer with HIF-β, which can bind to hypoxia responsive elements (HREs) in the promoters of hypoxia-regulated genes^10^.

Long noncoding RNAs (lncRNAs) are transcripts larger than 200 nucleotides that do not encode proteins and play multifunctional roles in modulating embryonic pluripotency, differentiation and development^11^. The lncRNA H19 is maternally expressed and paternally imprinted, and is located close to the telomeric region of chromosome 11p15.5^12^. H19 encodes a 2.3 kb noncoding RNA and is highly expressed in the developing embryo but is down-regulated neonatally in most tissues^13^. Recently, H19 has been described as an oncogene in various cancers such as bladder, colorectal, gastric and breast cancers^14^,^15^,^16^,^17^. Previously we performed a series of assays that demonstrated H19, which is highly expressed in glioblastomas, promotes glioma cell invasion by inducing miR-675 expression^18^. And it was reported for promoting angiogenesis, stemness and drug resistance of GBM^19^,^20^. The phenomenon of elevated H19 expression under hypoxia was studied in various cancer cells, and Hif-1α could promote H19 expression *in vitro*^21^. However, the relation of Hif-1α and H19 *in vivo* and in human clinical specimens remains unclear. And the mechanism through which Hif-1α supports upregulation of H19 level still requires further investigation.

In this study, we demonstrated that the mechanism of H19 induction under hypoxia is partly accounted for by Hif-1α directly binding to the H19 promoter, a process that requires specific protein 1 (SP1) to be activated by Hif-1α, which enhances H19 transcriptional activation in glioblastoma cells. Unexpectedly we found PTEN status is also an important factor to affect hypoxia-driven H19 level in multiple GBM cell lines and human clinical specimens. Moreover, hypoxia-induced H19 expression was found to promote a migratory and invasive phenotype by regulating the expression of epithelial-mesenchymal transition (EMT)-related proteins, including β-catenin^22^. H19 regulates β-catenin expression by binding mir-181d and inhibiting the miRNA repression of its target genes. This novel molecular mechanism demonstrated in our study contributes to a better understanding of glioblastoma pathogenesis and provides new therapeutic targets for malignant brain tumors.

## Results

### H19 expression is upregulated by Hif-1α under hypoxia

**Figure 1:**
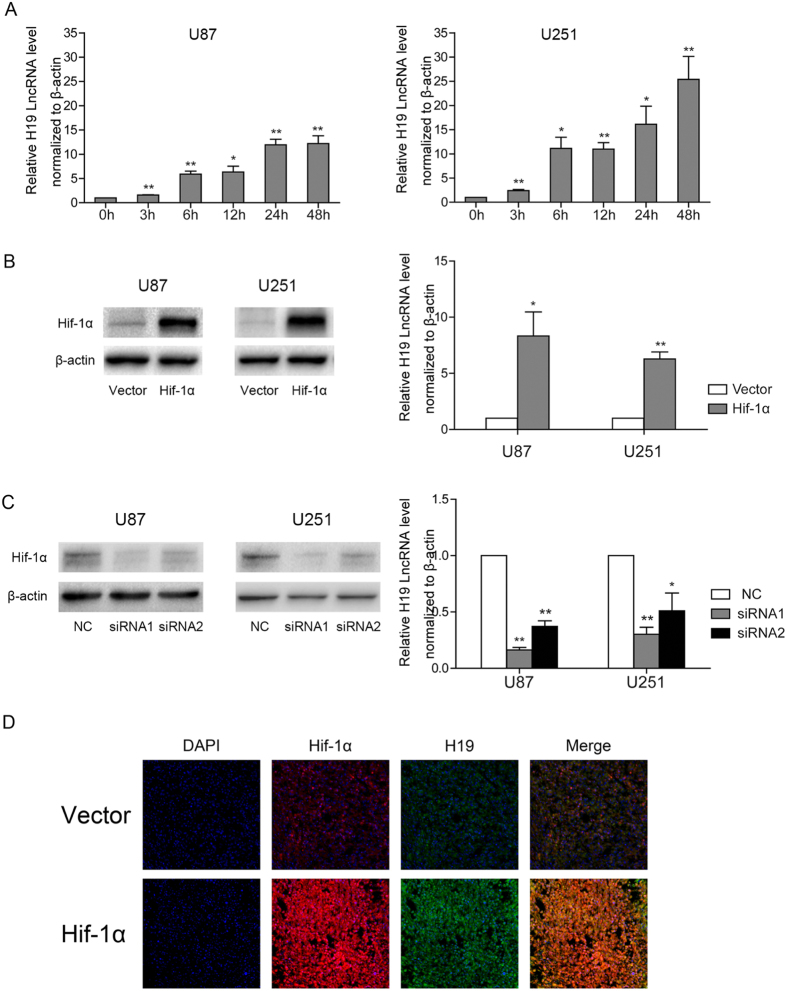
H19 expression is elevated under hypoxia and Hif-1α is a critical factor responsible for the induction of H19 RNA in U87 and U251 cells. The levels of H19 RNA normalized to β-actin were quantified by qPCR. Protein levels were detected by western blot and normalized to β-actin levels. (**A**) U87 and U251 cells were exposed to hypoxia (2% O_2_) for the indicated times. H19 levels at each time point (bar graphs) were normalized to normoxic controls. (**B**) U87 and U251 cells were transfected with Hif-1α expression plasmid or empty vector before culture under hypoxia for 24 h. (**C**) U87 and U251 cells were transfected with a control siRNA, or Hif-1α siRNA and cultured under hypoxia for 24 h. All experiments were repeated three times with similar results. (*p < 0.05, **p < 0.01). (**D**) Immunofluorescent double staining was performed on xenograft tumors after U87 cells stably expressing Hif-1α or control vector be subcutaneously injected. The positive expression of Hif-1α and H19 were overlapped in the same section of U87 xenograft tumors.

To explore the mechanisms underlying elevated H19 RNA levels in glioblastoma cells under hypoxia, cells were cultured in an anoxic condition (2% O_2_). As shown in Fig. 1A, qPCR analysis revealed a significant time-dependent increase of H19 RNA in U87 and U251 cells grown under hypoxia. Given that Hif-1α plays a critical role in the cellular response to hypoxia, we transfected U87 and U251 cells with a Hif-1α overexpression plasmid and again cultured the cells under hypoxia. Hif-1α overexpression under hypoxia produced a remarkable induction of H19 that was greater than hypoxia alone (Fig. 1B). Next, two different Hif-1α siRNAs were transfected into U87 and U251 cells. Both Hif-1α siRNAs decreased H19 induction under hypoxia (Fig. 1C). Re-expressing Hif-1α level in these cells knocked down Hif-1α expression recovered downregulated expression of H19 by Hif-1α siRNA (Fig. S1A). These results indicated that hypoxia induces H19 expression through Hif-1α in glioblastoma cells *in vitro*. Furthermore, Double-labelling immunofluorescence (IF) was performed to reveal Hif-1α-stimulated H19 in sections of xenograft stemmed from U87 cells transfected with control or Hif-1α expression lentivirus transplanted to nude mice. (Fig. 1D), which indicated that H19 is closely related to Hif-1α expression *in vivo*.

### PTEN regulates H19 expression under hypoxia

**Figure 2:**
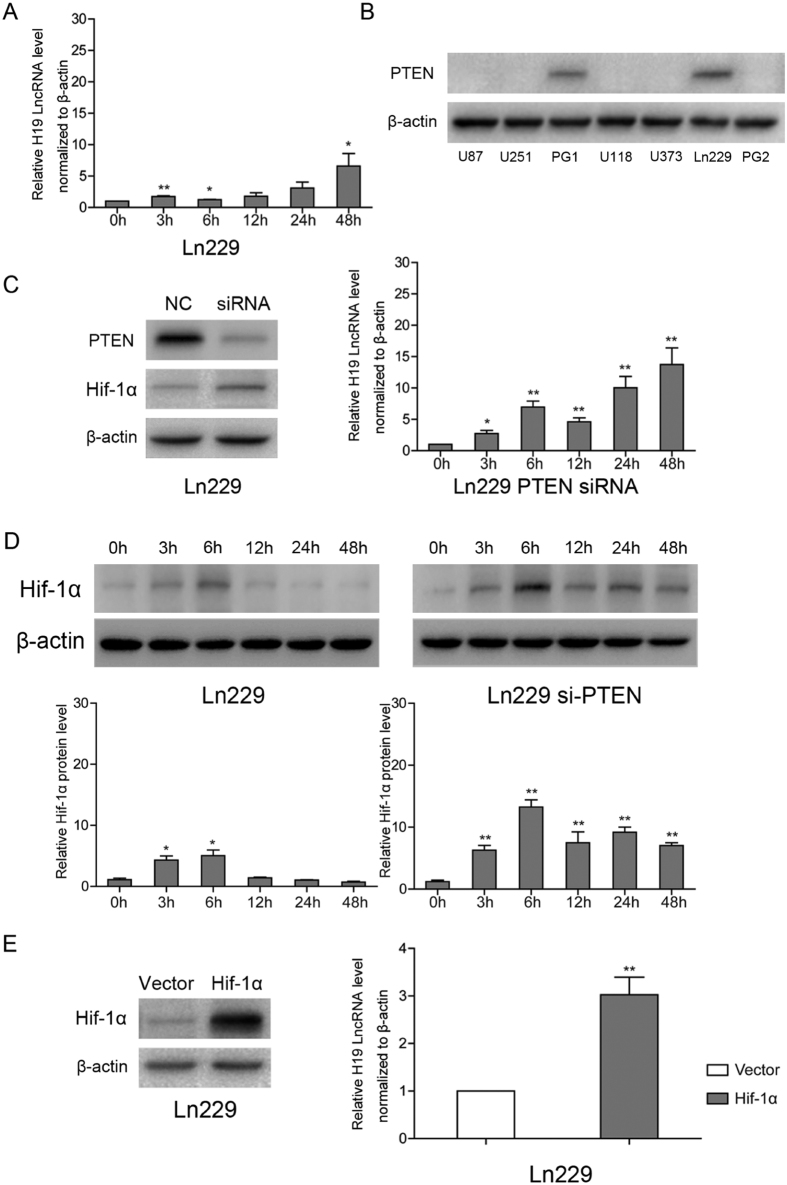
H19 induction is dependent on PTEN status at the early stages of hypoxia in Ln229. PTEN interferes with H19 induction by promoting Hif-1α degradation under hypoxia. (**A**) Ln229 cells were exposed to hypoxia (2% O_2_) for the indicated time periods. (**B**) PTEN protein levels analyzed by western blot in U87, U251, Ln229, U373, U118, GP1 and GP2 cells. (**C**) PTEN siRNA and control siRNA treatment of Ln229 cell before hypoxic cultivation. Western blots show that PTEN inhibited Hif-1α expression in Ln229 cells under hypoxia at 6 h. (**D**) Hypoxia induced different effects on Hif-1α protein levels between control siRNA-treated Ln229 cells and PTEN siRNA-treated Ln229 cells. (**E**) Ln229 cells transfected with an exogenous Hif-1α expression plasmid or an empty vector. All experiments were repeated three times with similar results. (*p < 0.05, **p < 0.01).

Interestingly, H19 expression remained low after 48 h culture under hypoxia in Ln229 glioblastoma cells (Fig. 2A). One possible explanation for this phenotype is the different genotypes of the cell lines. It has been reported that PTEN loss can affect Hif-1α stability under hypoxia through PI3K/Akt pathway activation in glioblastoma-derived cell lines^23^. Furthermore, PTEN status in the three cells was different; U87 cells are PTEN null, U251 cells have mutated PTEN and PTEN is wild-type in LN229 cells. To investigate the role of PTEN in influencing H19 level under hypoxia, we analyzed multiple GBM cell lines including two primary GBM cells (GP1 and GP2) collected from two patients. Western blot assays indicated significantly lower PTEN protein expression in U87, U251, U373, U118 and GP2 cells compared with Ln229 and GP1 cells (Fig. 2B). Further, the results showed that the significant elevated H19 level under hypoxia was not observed in cells with normal level of PTEN, but in cells with very low level of PTEN (Fig. S1B).

Then we demonstrated that H19 expression in response to hypoxia was elevated in Ln229 cells after PTEN siRNA treatment (Fig. 2C). The opposite result was observed in U87 cells treated with exogenous PTEN plasmid (Fig. S1C). We next explored Hif-1α dynamics at different time points under hypoxia. Hif-1α protein achieved maximum expression after 6 h under hypoxia and was then quickly degraded in Ln229 cells. Contrarily, Hif-1α maintained relatively stable expression after 6 h time point in PTEN siRNA-treated Ln229 cells (Fig. 2D). Finally, H19 induction was also stimulated by Hif-1α overexpression (Fig. 2E). Taken together, PTEN could impact the stability of Hif-1α thereby influencing H19 level under hypoxia.

### The expression of Hif-1α and H19 in human brain tissues

**Figure 3:**
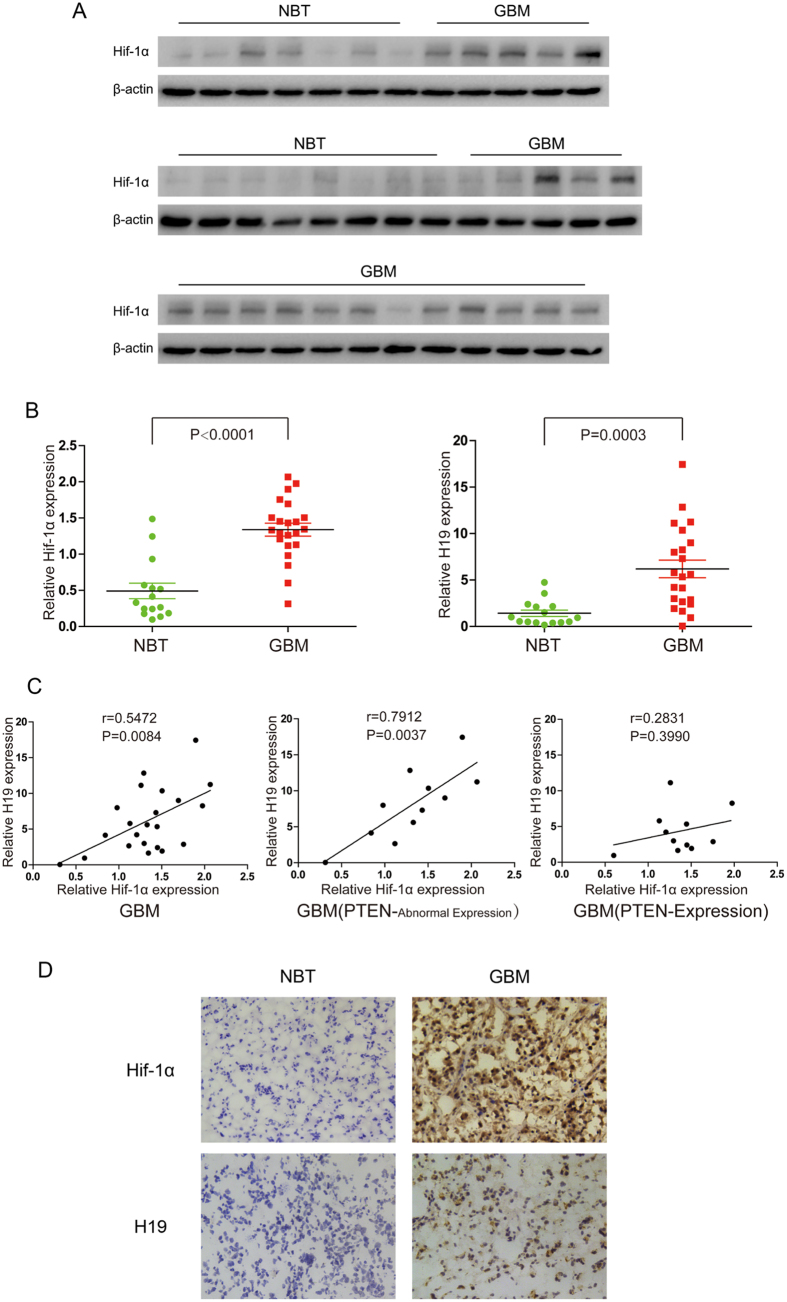
Expression levels of Hif-1α and H19 in human glioblastoma (GBM) and adjacent normal brain tissues (NBT). (**A**) The levels of Hif-1α in GBM and NBT were detected by western blot. The expression of Hif-1α was normalized to β-actin. (**B**) The levels of H19 in GBM and NBT were detected by QPCR. The expression of H19 was normalized to β-actin. (**C**) The correlation between Hif-1α and H19 in GBM specimens or in the subgroup on the basis of PTEN status. Pearson’s correlation coefficient was applied. (**D**) Immunohistochemistry analysis of Hif-1α and H19 in two representative tissues (one NBT specimen and one GBM specimen).

The expression of Hif-1α, PTEN and H19 levels were analyzed in 22 human glioblastoma tissues (GBM) and 15 adjacent normal brain tissues (NBT) removed from patients. As shown in Fig. 3A and B, Upregulation of Hif-1α in human GBM was significantly obvious relative to NBT, which were analogous to the results of H19 between GBM and NBT. The Fig. S1D showed different PTEN status in 22 GBM specimens, since the negative expression of PTEN is prevailing in GBM^24^. Further the correlation between Hif-1α and H19 expression was analyzed in human GBM specimens. The Hif-1α levels was positively correlated with H19 levels in GBM specimens (r = 0.5472, P < 0.01). Moreover, the Pearson’s correlation is up to 0.7912 (P < 0.01) in PTEN-abnormal expression group of GBM samples, but only 0.2831 (P = 0.399) in PTEN-expression group (Fig. 3C).

Then Hif-1α and H19 levels were detected immunohistochemically in two representative specimens including NBT and GBM tissues. Figure 3D demonstrated that Hif-1α was highly detected in nuclei and cytoplasm of cancer cells but rarely expressed in normal brain tissues, whereas H19 was concentrated in the cytoplasmic area of GBM tissues compared to the very low level in normal brain tissues. These results further support that Hif-1α positively correlates with H19 in human GBM tissues. And PTEN status is an important factor beneficial for the correlation of Hif-1α and H19.

### Hif-1α promotes H19 expression by directly binding to the H19 promoter

**Figure 4:**
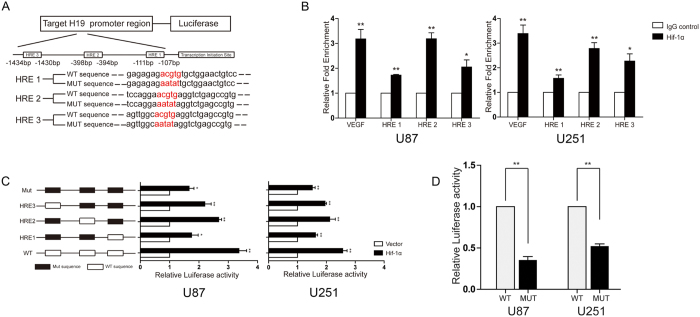
Hypoxia response elements (HREs) in the H19 promoter are not the only mechanism to mediate Hif-1α-induced H19 expression. (**A**) Sequences and locations of the three HREs within 2 kb before the transcription start site in the H19 promoter. The wild-type HRE consensus sequence of designed luciferase report construct was 5′-ACGTG-3′, and the mutant HRE sequence was 5′-AATAT-3′. (**B**) Chromatin immunoprecipitation assays were performed to indicate binding of Hif-1α to the HREs located in the H19 and VEGF promoters. Mouse anti-Hif-1α antibody or control mouse IgG was used for immunoprecipitation with DNA isolated from U87 and U251 cells. The immunoprecipitate was amplified by qPCR using primers targeting the HREs. The results were normalized to the negative control IgG. The HRE in the VEGF promoter was used as positive control. (**C**) Five luciferase reporter plasmids were designed containing different combinations of wild type and mutated HREs (schematized on the left). Relative luciferase activity was observed for each reporter construct following transfection of empty vector or Hif-1α (horizontal bars, right) in U87 and U251 cells with 24 h hypoxia treatment. Renilla activity was used as an internal loading control. (**D**) The relative luciferase activity for each reporter construct following transfection with the same Hif-1α plasmid normalized to the WT group. (*p < 0.05, **p < 0.01).

Through sequence analysis of the human 5′-H19 promoter region, we found three putative Hif-1α binding sites, named HRE1, 2 and 3, within approximately 2000 bp upstream of the transcription start site (Fig. 4A). To understand the role of these HREs on promoter-driven expression of H19, ChIP assays were performed to verify an interaction between Hif-1α and the H19 promoter in U87 and U251 cells. We used the HRE in the VEGF promoter as positive control, as VEGF is a well-documented direct transcriptional target of Hif-1α. Using primers targeting HREs in the H19 promoter for ChIP-qPCR, we found a mild enrichment of H19 HREs immunoprecipitated by Hif-1α compared with negative control IgG (Fig. 4B). Five luciferase reporter plasmids containing segments of the 2000 bp 5′-flanking region of the H19 promoter were then synthesized (Fig. 4A). Interestingly, in both U87 and U251 cells, Hif-1α expression resulted in increased luciferase activity in all groups compared to the negative control plasmid, even when all HRE sequences were mutated (Fig. 4C). We then analyzed luciferase activity in cells transfected with the same quantity of Hif-1α expression plasmid but with different report genes. There were statistical differences between mutating all HRE sites compared to the reporter construct with all HREs not mutated (Fig. 4D). This result with that in Fig. S5E demonstrated that Hif-1α at least partly promoted H19 level via directly binding sites in the H19 promoter.

### SP1 assists Hif-1α-dependent H19 expression by directly binding to the H19 promoter

As shown in Fig. 4D, even when all HREs were mutated, Hif-1α expression induced luciferase activity from the H19 reporter construct, suggesting Hif-1α may elicit H19 promoter-driven expression through another pathway as well as through direct transcriptional regulation.

**Figure 5:**
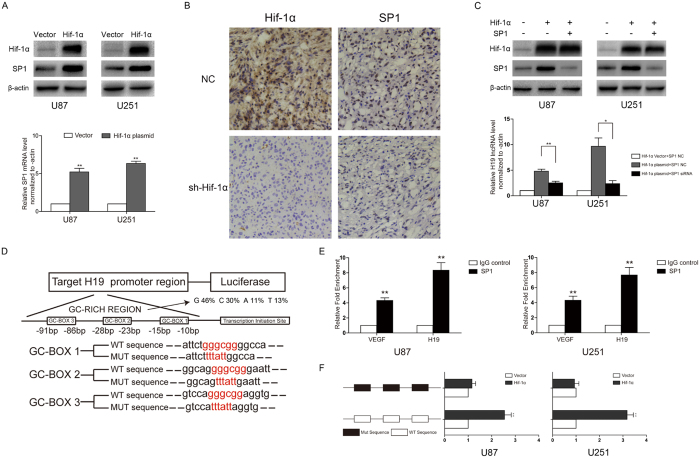
Hif-1α indirectly stimulates H19 expression through SP1 regulation and SP1 regulates H19 by directly binding to the SP1 binding sites in the H19 promoter region in U87 and U251 cells. (**A**) U87 and U251 cells transfected with the Hif-1α expression plasmid or empty vector and cultured under hypoxia for 24 h. (**B**) IHC analysis of consecutive sections of Hif-1α and SP1 expression in subcutaneous xenografts originated from U87 cells after Hif-1α sh-RNA treatment. (**C**) U87 and U251 cells co-transfected with the Hif-1α expression plasmid and SP1 siRNA and cultured under hypoxia for 24 h. (**D**) The locations of the putative SP1 binding sites within 100 bp upstream of the transcription start site in the H19 promoter. These binding sites constituted a GC-rich region. The wild-type sequence of reporter construct was 5′-GGGCGG-3′, and the mutant one was 5′-TTTATT-3′. (*p < 0.05, **p < 0.01). (**E**) ChIP analysis confirmed SP1 directly bound to the H19 promoter through the three GC-boxes. GC-boxes from the VEGF promoter were used as positive controls. (**F**) Two reporter plasmids harboring wild type or mutant GC-boxes were co-transfected with the SP1 expression plasmid or empty vector and the Renilla plasmid into U87 and U251 cells. Relative luciferase activity was analyzed after 24 h treatment. (*p < 0.05, **p < 0.01).

SP1 has also been shown to mediate the transcriptional response to hypoxia, and recently a study confirmed that elevated SP1 protein levels could drive binding of Hif-1α to the HREs within the SP1 promoter^25^. Q-PCR and western blotting demonstrated that SP1 mRNA and protein were upregulated by Hif-1α (Fig. 5A). A similar result obtained *in vivo* study was displayed by immunohistochemistry (IHC) examination (Fig. 5B). Hypoxia-induced H19 expression was also blocked when cells were transfected with SP1 siRNA (Fig. S2A). And knocking down SP1 expression also cause downregulation of H19 *in vivo* by U87 cells with double-staining IF (Fig. S2B). Furthermore, a rescue experiment was performed in which U87 and U251 cells were co-transfected with a Hif-1α expression plasmid and SP1 siRNA. As shown in Fig. 5C, the upregulation of H19 RNA by Hif-1α was disrupted by SP1 knockdown. The opposite results were observed when Hif-1α siRNA was co-transfected with an SP1 expression plasmid in U87 and U251 cells (Fig. S2C). Thus, SP1 plays a pivotal role along with Hif-1α in H19 regulation under hypoxia.

SP1 often binds to GC-rich motifs, also known as GC-box elements, of many promoters to enhance gene transcription. We found a length of GC-rich sequence within 100 bp upstream of the transcription start site in H19 promoter (Fig. 5D). The proportions of G and C are 46% and 30% respectively, while A and T are only 24% together. ChIP assays were then performed to examine SP1 binding to the H19 promoter. The SP1 binding site in the VEGF promoter was used as positive control because VEGF is a direct target of SP1. The binding efficiency of SP1 to the H19 promoter was even stronger than to the VEGF promoter (Fig. 5E). Two luciferase reporter plasmids were designed harboring at least 1 kb of the H19 promoter region including a wild-type or mutated GC-box sequence (Fig. 5D). After transfecting the SP1 expression plasmid, luciferase activity in wild type group was significantly elevated compared to the negative control group. But in the mutated GC-box group no statistical difference was observed between the SP1 overexpression and control group (Fig. 5F). Together, these data show that SP1 binds strongly to the H19 promoter region through its specific binding sites (GC-boxes) and that this interaction is required for optimal H19 transcriptional activation.

In addition, to validate the specificity role of SP1 in Hif-1α-dependent H19 level, two H19 report genes were constructed including a wild-type or mutated SP1 binding sequence with all mutated HRE binding sites. The result in Fig. S2D showed the ascending luciferase activity of H19 report gene with all mutated HRE sites was mainly attributed to SP1 binding site, since introducing the exogenous Hif-1α level can stimulate the endogenous SP1 expression (Fig. 5A). Besides, we analyzed the effects of Hif-1α on regulation of H19 level without participation of SP1. The result in Fig. S2E demonstrated even without SP1 binding to H19 promoter, the exogenous Hif-1α level still could stimulate luciferase activity of the report gene including wild-type HREs in H19 promoter, rather than that including mutated HREs. This result also supported that Hif-1α could promote H19 expression by itself.

### H19 overexpression confers a poor prognosis for GBM patients and regulates hypoxia-induced malignant functions

**Figure 6:**
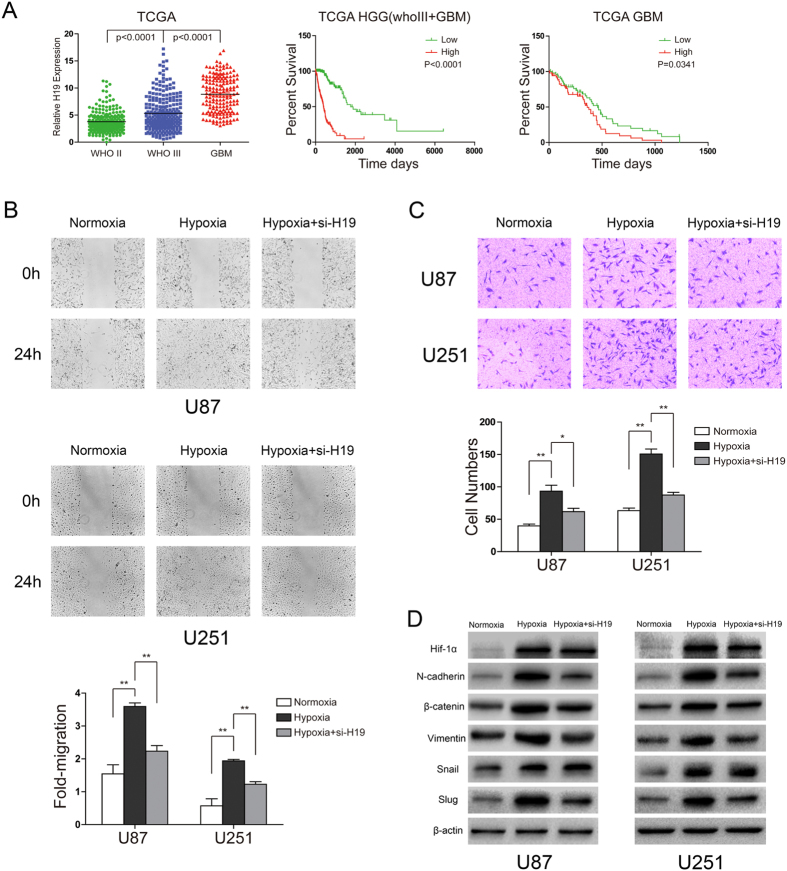
H19 plays an important role in hypoxia-driven migration and invasion in U87 and U251 cells. H19 regulates EMT-related protein expression, affecting migration and invasion in U87 and U251 cells. U87 and U251 cells were transfected with control siRNA or H19 siRNA. The samples are derive from the same experiment and that blots were processed in parallel. (**A**) Increased H19 expression correlates with glioma grade and confers a poor prognosis in high grade glioma (HGG) and GBM patients. Levels of H19 were analyzed in different glioma tissues of TCGA data. Kaplan–Meier survival curves for H19 expression in HGG and GBM patients of TCGA data. (**B**) The scratch-wound gap was photographed before and after 24 h incubation under normoxia and hypoxia. After performing the wound healing test. The results were analyzed by measuring the range of migrating cells from 3 different fields for each wound. (**C**) Transwell migration assays were used to evaluate glioblastoma cell invasion. Migrated cells with normoxic or hypoxic treatment for 24 h were stained with crystal violet and counted for statistical analysis. (**D**) EMT-related proteins in the three cell groups were examined by western blot and normalized to β-actin. (*p < 0.05, **p < 0.01). The samples are derived from the same experiment and that blots were processed in parallel.

The oncogenic effects of H19 in glioblastomas were explored by public database of expression. H19 was significantly upregulated in glioblastomas compared to low grade gliomas in The Cancer Genome Atlas (TCGA) (Fig. 6A). Next the decreased survival were associated with specimens expressing high levels of H19 than those with low H19 levels by Kaplan–Meier survival curve analysis from primary high grade glioma (WHOIII+GBM) and GBM patients only in TCGA database with full survival data (the Log-Rank test P for HGG < 0.0001 and the Log-Rank test P for GBM = 0.0341) (Fig. 6A). Since high levels of H19 have a markedly poor outcome in glioblastomas, glioblastoma cells cultured under hypoxia showed a stronger migration and invasion capacity than cells cultured under normoxia, these enhanced capacities could be relieved by knocking down H19 (Fig. 6B,C). Furthermore, EMT-related proteins, such as N-cadherin, β-catenin, Vimentin, Snail and Slug were upregulated under hypoxia, since EMT-related proteins is considered to regulate migration and invasion in cancers, including mesenchymal-like transition in malignant gliomas^26^,^27^ (Fig. 6C). When H19 was knocked down, expression of EMT-related proteins except Snail showed a significant reduction.

### H19 antagonizes miR-181d blocking β-catenin repression

**Figure 7:**
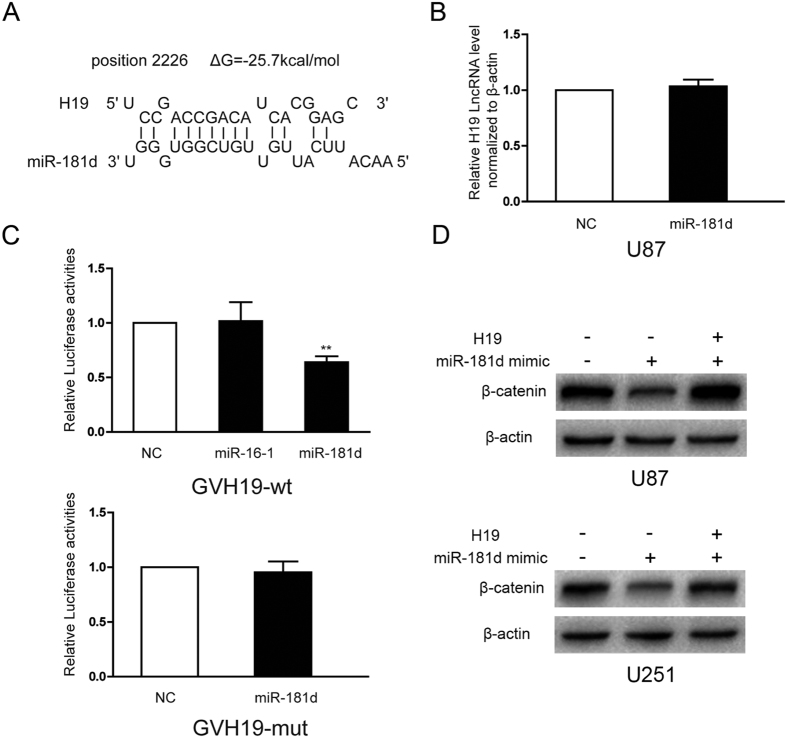
H19 affects β-catenin expression by regulating miR-181d activity. (**A**) Bioinformatics predicted a miR-181d binding site in human H19. (**B**) Ectopic miR-181d expression by miRNA mimics did not affect the H19 levels in U87 cells. (**C**) U87 cells were co-transfected with miR-181d mimics or miR-16-1 mimics and luciferase reporters harboring the miR-181d binding site sequence or a mutated one from H19. Luciferase activities were normalized to the activities in cells expressing scrambled miRNA mimics. (**D**) U87 and U251 cells were co-transfected with miR-181d mimics and H19 expression plasmids, β-catenin protein levels were examined by western blot. (*p < 0.05, **p < 0.01). The samples are derived from the same experiment and that blots were processed in parallel.

H19 has been shown to bind and inhibit the activity of miRNAs, affecting target gene expression in tumor cells; therefore, we hypothesized that this may be the mechanism through which H19 regulates the expression of EMT-related proteins. Using RNAHYBRID Bioinformatic tools, we found that H19 contained a putative binding site for miR-181d, which is known to directly target β-catenin^28^ (Fig. 7A). As shown in Fig. 7B, miR-181d mimics did not affect H19 expression by qPCR. After transfecting a luciferase reporter plasmid harboring the putative miR-181d binding site from H19, the relative luciferase activity decreased in cells transfected with miR-181d mimics but not miR-16-1 mimics, the miRNA could not combine with H19 as previously reported^29^. After transfection with a reporter gene containing mutated binding sites for miR-181d, the reduced luciferase activity was not observed with miR-181d mimics (Fig. 7C). These data suggest that the putative miR-181d binding site is essential for the interaction of miR-181d and H19 RNA. Furthermore, miR-181d overexpression decreased the β-catenin levels, and H19 upregulation facilitated β-catenin accumulation by aborting the inhibition of miR-181d in both U87 and U251 cells (Fig. 7D). These data confirmed that H19 acts as a molecular sponge for miR-181d that abolishes miR-181d inhibition of β-catenin.

## Discussion

Besides protein coding RNAs, it is clear that non-coding RNAs are also differentially expressed under hypoxia and play major roles in the hypoxic tumor microenvironment; a host of cellular miRNAs were recently discovered to be upregulated in tumors during hypoxia. Specifically, miR-210 has been proven to be significantly upregulated under hypoxia in patient-derived glioblastoma spheroids^30^, and Hif-1α was shown to be the dominant force for miR-210 overexpression^31^. The lncRNAs induced by hypoxia in tumors have also been published recently. HIF-2α has been shown to bind to sequences upstream of the NEAT1 lncRNA promoter in MCF-7 breast cancer cells^32^. Hif-1α specifically binds two HREs in the UCA1 lncRNA promoter, leading to its activation in hypoxic bladder cancer cells^33^. Our study demonstrated that H19 RNA is upregulated under hypoxia and its expression is augmented over time in GBM cells with very low level of PTEN, but H19 was induced less in PTEN-expression cells. It has been reported that PTEN regulates the E3 ligase MDM2 to promote Hif-1α degradation in a proteasome-dependent manner^34^. Our results revealed PTEN protein level is prominent in Ln229 and GP1 cells, but very low or absent in U251, U87, U373, U118 and GP2 cells. We found that decreasing PTEN protein levels could maintain Hif-1α expression and elevate H19 RNA levels under hypoxia in Ln229 glioblastoma cells, and vice versa in U87 cells. We also proved significantly positive correlation of Hif-1α and H19 depending on PTEN status in human GBM, since mutation or loss of expression of PTEN is usual in glioma and occurs in about 40% of GBM^24^. These results showed Hif-1α actively modulated H19 *in vitro* and *in vivo*, and PTEN is an advantageous condition for hypoxia-driven H19 induction.

Furthermore, our study demonstrated both a direct and indirect regulation H19 RNA expression by Hif-1α. Several studies have shown that Hif-1α enhances HRE-driven transcriptional activation in conjunction with adjacent SP1 binding sites (GC-boxes). For example, CD147 upregulation is mediated by the combined effects of Hif-1 and SP1 under hypoxia in epithelial tumors^35^. The hypoxia-responsive multidrug resistance gene (MDR1) is also positively modulated by both Hif-1α and SP1 in tumors^36^. Besides the collaboration of Hif-1α and SP1 in enhancing hypoxia-induced gene transcription, the sequential activation of SP1 by Hif-1α under hypoxia has also been reported for genes activated by hypoxia. For example, Hif-1α induces SP1 expression, which regulates expression of the prion protein PRNP in cancer^37^. A recent report indicated that Hif-1α binds directly to HREs in the SP1 promoter to increase SP1 levels in cerebral ischemia^25^. We verified that Hif-1α can promote SP1 mRNA and protein expression under hypoxia, and that SP1 has a critical intermediary role in Hif-1α-driven H19 expression, binding GC-boxes in the H19 promoter.

Finally, we showed that the mechanism through which H19 regulates glioblastoma migration and invasion involved β-catenin expression. Some lncRNAs generate miRNAs to control gene expression, such as miR-206 and miR-133b, which are derived from the lncRNA Linc-MD1 during muscle differentiation^38^. It has emerged recently that lncRNAs can also regulate miRNAs biogenesis by acting as a molecular sponges that reduce the amount miRNAs available to interact with mRNA targets. Such has been reported that H19 regulates muscle differentiation through this mechanism, acting as an antagonist complementary with let-7e, let-7g, and let-7i^29^. In colorectal cancer H19 interacts with miR-200a and miR138 to boost Vimentin and ZEB1 expression^39^. Since miR181d inhibits β-catenin expression by directly binding the 3′-UTR of the β-catenin message^28^, and β-catenin acts as an intracellular signal transducer in the Wnt signaling pathway involved in EMT-like in GBM^40^, we verified H19 sponge activity towards miR-181d affects β-catenin levels in U87 and U251 cells. This novel mechanism offers a new understanding of how H19 and miRNAs interact to control gene transcription and offers a new view of the oncogenic role of H19 in glioblastomas.

In a summary, we found that Hif-1α directly promoted H19 expression through binding to the H19 promoter and indirectly through SP1-mediated H19 transcriptional activation under hypoxia in glioblastoma cells. Moreover, H19 expression upregulated β-catenin expression via binding and inhibiting miR-181d, which negatively regulated β-catenin mRNA, thereby contributing to H19 oncogenic functions in glioblastomas. These findings reveal the essential role of H19 during hypoxia in glioblastoma formation and progression and suggest H19 activity could serve as the potential target for glioma therapy.

## Methods

### Cell lines and hypoxia treatment

The human glioma cell lines U87, U251, Ln229, U373 and U118 were purchased from the Chinese Academy of Sciences Cell Bank (Shanghai, China). A primary culture designated GBM1 (GP1) was established in February 2016 from the tumor cells of a patient with a left frontal glioblastoma, and a culture designated GBM2 (GP2) was established in March 2016 from tumor cells taken from a patient with a left tempus glioblastoma. Tumor tissue was collected from a patient who granted written informed consent. The Institutional Review Board of the First Affiliated Hospital of Nanjing Medical University approved the study protocol.

Tissue was obtained from regions comprising viable tumor cells. Within 2 h after acquisition, the tissue samples were dissociated into single-cell suspensions, washed with Hanks solution (Solarbio, Beijing, China) to remove red blood cells, and the number of cells was counted. All cell lines were cultured at 37 °C in 5% CO_2_ in Dulbecco’s modified Eagle’s medium (DMEM) (Hyclone Laboratories, Utah, USA) supplemented with 10% fetal bovine serum (FBS) (Gibco, CA, USA). Hypoxia was achieved using a hypoxia incubator HEPA filter (Thermo Fisher Scientific, Boston, USA) flushed with 2% O_2_, 5% CO_2_ and 93% N_2_.

### Human brain tissues

Human glioblastoma specimens and normal brain tissues were obtained from The First Affiliated Hospital of Nanjing Medical University. The normal brain tissue samples were taken from adjacent glioblastoma tissues.

### Study approval

The use of human gliomas and adjacent normal tissues were approved by Research Ethics Committee of Nanjing Medical University (Nanjing, Jiangsu, China) and were performed in accordance with the approved guidelines. The evaluation of the Hif-1α and H19 level of the tissues was in accordance with the approved guidelines. Informed consents were obtained from the patients.

### RNA extraction and quantitative real-time PCR

RNA extraction was performed from human tissue samples or cell lines using TRIzol reagent (Life Technologies, CA, USA) following the manufacturer’s protocol. Quantitative real-time PCR was conducted using the ABI StepOne Plus system (Applied Biosystems, CA, USA) with TaqMan-based real-time reverse Transcription Kit (Thermo Fisher Scientific) and SYBR Green quantitative Real-Time PCR Master Mixes (Roche, Basel, Switzerland) to detect H19 or other RNA species. Primers were purchased from Invitrogen (Shanghai, China) and are shown in Table 1. Data were analyzed by the 2^−ΔΔCt^ method and results were normalized to β-actin expression.

### Oligonucleotides and transfection conditions

The miR-181d, miR-16-1 and scrambled miRNA mimics were chemically synthesized by Ribo (Guangzhou, China), and small interfering RNA (siRNA) oligonucleotides targeting Hif-1α, SP1 and PTEN were chemically synthesized by GenePharma (Shanghai, China). The sequences of these reagents are shown in Table 1. Cells were transfected 24 h after plating using Lipofectamine 2000 (Invitrogen). Transfection complexes were prepared according to the manufacturer’s instructions and added directly to the cells in Opti-MEM reduced serum media (Gibco).

### Lentivirus packaging and stable cell lines

Lentivirus carrying Hif-1 or vector controls, sh-Hif-1α, sh-SP1 or sh-RNA control were designed and packaged by Genechem (Shanghai, China). Lentivirus were packaged in HEK-293T cells and collected from the medium supernatant. Stable cell lines were established by infecting lentivirus into U87 cells and selected by puromycin.

### Vector construction and luciferase reporter assay

The 5′-flanking regions of the human H19 promoter (NG_016165) (2000 or 1000 bp upstream of the transcription start site) containing wild type or mutated HREs (5′-RCGTG-3′) and SP1-binding sites (5′-GGGCGG-3′) were chemically synthesized and cloned into reporter plasmids (GV238- and GV264-basic). The miR-181d binding site from the H19 lncRNA (NR_002196), along with a mutated version of the sequence, was also chemically synthesized and cloned into a reporter plasmid (GV306-basic). All reporter plasmids as well as expression plasmids for Hif-1α (NM_001530), SP1 (NM_138473), H19 (NR_002196) PTEN (NG_007466) were designed by Genechem (Shanghai, China) with primers listed in Table 1. Cells cultured in 24-well plates were co-transfected with luciferase reporter plasmids, target gene expression plasmids or miRNA mimics as well as the internal control Renilla plasmid. After 48 h, luciferase activity was analyzed using the Dual Luciferase Reporter Assay System (Promega, WI, USA) according to the manufacturer’s protocol.

### Western blotting

Total protein was extracted from human glioma cells or human brain tissues with RIPA lysis buffer (KenGEN, Hong Kong, China) and were quantified using the bicinchoninic acid protein assay kit (KenGEN). Equal amounts of protein lysate (30 μg) from each sample were subjected to SDS-PAGE electrophoresis and transferred to nitrocellulose membranes (Merck, NJ, USA). After blocking in 5% non-fat milk, membranes were incubated overnight at 4 °C with diluted (1:500) primary antibodies against Hif-1α, SP1 (Abcam, Cambridge, UK), PTEN, N-cadherin, β-catenin, Vimentin, Snail, Slug and β-actin (Cell Signaling Technology, MA, USA), followed by incubation with a horseradish peroxidase-conjugated secondary antibody (1:2000, Santa Cruz Biotechnology, CA, USA) for 2 h. After washing with PBST, membranes were probed using SuperSignal^®^ Maximum Sensitivity Substrate (Thermo Fisher Scientific). The image processing was implemented by the software image lab 4.0.

### Chromatin immunoprecipitation (ChIP) assays

ChIP assays were performed with technical support from Genecreate (Wuhan, China). Chromatin was prepared and immunoprecipitated using mouse anti-Hif-1α and rabbit anti-SP1 antibodies (Abcam). Negative control samples were prepared using control rabbit IgG antibody. Immunoprecipitated chromatin was analyzed by QPCR using primers targeting individual HREs and GC-boxes in the human H19 promoter. The HRE and GC-box from the VEGF promoter were chosen as positive controls. The primer sequences used for ChIP-qPCR are listed in Table 1. PCR conditions were set according to the instructions provided in SYBR Green Kit (Roche). Results were analyzed as previously described^41^.

### *In vitro* cell migration and invasion assays

A wound-healing assay was used to analyze cell migration. Oligonucleotides were transfected into cells in 6-well plates, and after 24 h, wounds were created with a sterile pipette tip on the confluent cell monolayer. The cells were then cultured under normoxia or hypoxia for 24 h, and images of the wound width were taken time 0 and 24 h. The transwell assay was used to quantitate cell invasion; 3 × 10^4^ cells were transfected with the indicated oligonucleotides and transferred atop Matrigel-coated invasion chambers (BD Biosciences, CA, USA) in a serum-free DMEM. DMEM containing 10% FBS was added to the lower chamber simultaneously. After 24 h culture under normoxia or hypoxia, noninvasive cells were removed, and the invasive cells were fixed with 4% paraformaldehyde, stained with 0.1% crystal violet, and imaged (Magnification: 100×). The presented data represent three independent experiments.

### Xenograft mouse model

Animal experiments were approved by the Animal Management Rule of the Chinese Ministry of Health (documentation 55, 2001) and were in accordance with the approved guidelines and the experimental protocol of Nanjing Medical University. U87 cells (1 × 10^6^) stably expressing negative control or treatment group genes were subcutaneously injected into 5-week-old female nude mice (Cancer Institute of the Chinese Academy of Medical Science). Fifteen days after injection, the nude mice were sacrificed and the tumor tissues were excised and frozen immediately at −80 °C for further study in case of the degradation of RNA and Hif-1α.

### Fluorescence *in situ* hybridization (FISH)

RNA FISH was performed as described previously elsewhere with minor modifications^42^. The probes of *in situ* hybridization for H19 were synthesized from GoodBio (Wuhan, China). The fresh tissues fixed in 4% formaldehyde for 1 h, then dehydrated in 15% Sucrose solution for 8 h. The tissues were fixed in 4% formaldehyde for 10 min, washed by PBS (PH7.4) for 5 min three times, digested by protease K for 2 min, washed again by PBS (PH7.4) for 5 min three times. After eliminating auto-fluorescence and closing endogenous biotin, the sections were hybridized with probes for overnight. Then, tissue sections were washed with pre-warmed 2 × SSC at 37 °C for 10 min, 1 × SSC at 37 °C for 10 min, 0.5 × SSC for 10 min. After being closed in BSA for 30 min at room temperature, tissue sections were added 488-avidin (1:400) and incubated at room temperature for 50 min, washed 4 times with PBS for 5 min. Tissue sections were incubated in immune first antibody for overnight and in second antibody corresponded species of first antibody for 50 min at 4 °C. Finally tissue sections were washed twice with PBS and mounted on a medium containing DAPI.

### Immunohistochemistry

Briefly, fresh specimens were under cryopreservation and routinely processed into frozen sections. Five-micron-thick sections were prepared, and immunohistochemical staining with streptavidin-biotin immunoperoxidase assay was performed using antibodies against Hif-1α, SP1 (1:100 abcam) and probe of *in situ* hybridization for H19 (GoodBio, Wuhan, China). Slides were imaged under a light microscope (Leica, German) at 200× magnification. The expression level of examined protein was scored on a scale of 0 to 3 (0, negative; 1, slight positive; 2, moderate positive; 3, intense positive).

### Statistical analysis

All values are presented as mean + SD, and statistical analyses were performed with the Student’s t-test for differences in each two-group comparison, while one-way ANOVA was used to determine the difference among at least three groups using SPSS v19.0 for Windows. (SPSS, IL, USA). Pearson’s correlations analysis and Kaplan–Meier survival analysis were done employing GraphPad 5.0 software. P < 0.05 indicates a significant difference.

## Additional Information

**How to cite this article:** Wu, W. *et al*. Hypoxia induces H19 expression through direct and indirect Hif-1α activity, promoting oncogenic effects in glioblastoma. *Sci. Rep.* **7**, 45029; doi: 10.1038/srep45029 (2017).

**Publisher's note:** Springer Nature remains neutral with regard to jurisdictional claims in published maps and institutional affiliations.

## Supplementary Material

Supplementary Figures

## Figures and Tables

**Table 1:** Oligonucleotide sequence of PCR primers,siRNA fragments and expression plasmid.

Primers for qRT-PCR	Sequence-forward	Sequence-backward
H19	5′-AGAAATGGTGCTACCCAGCTC-3′	5′-CTGTTCCGATGGTGTCTTTGAT-3′
SP1	5′-TGGCAGCAGTACCAATGGC-3′	5′-CCAGGTAGTCCTGTCAGAACTT-3′
β-actin	5′-TCACCCACACTGTGCCCATCTACGA-3′	5′-CAGCGGAACCGCTCATTGCCAATGG-3′
**Primers for ChIP**	**Sequence-forward**	**Sequence-backward**
HRE 1 (-111/-107)	5′-AAACATCCCAGGTCATCCAAGC-3′	5′ -CCCTAGTATCTCCTCCCATCTCC-3′
HRE 2 (-398/-394)	5′-CACGCTCAGGGATCATCACG-3′	5′-TGTGGGCAAATTCACCTCTCC-3′
HRE 3 (-1434/-1430)	5′-GCACCCCACCCCTACTCTCC-3′	5′-CCACCATCACGGCTCAGACC-3′
VEGF HRE (-509/-505)	5′-TGAGTGAGTGTGTGCGTGTG-3′	5′-ATCTATTGGAATCCTGGAGTGACC-3′
SP1 GC-BOXES (-10/-91)	5′-ATGGGTGCTGGGTGAGAGAG-3′	5′-CCCTGCTCCTCGGTCCTAG-3′
VEGF GC-BOX (-234/-238)	5′-CTCCCTCCTCGCCAATGCC-3′	5′-GCCTCAGCCCTTCCACACG-3′
**siRNA for target genes**	**Sequence-sense**	**Sequence-anti-sense**
HIF-1α siRNA1	5′-GGCGAAGUAAAGAAUCUGAtt-3′	5′-UCAGAUUCUUUACUUCGCCtt-3′
HIF-1α siRNA2	5′-GUAGCCUCUUUGACAAACUtt-3′	5′-AGUUUGUCAAAGAGGCUACtt-3′
SP1 siRNA	5′-GAGUCACCCAAUGAGAACAtt-3′	5′-UGUUCUCAUUGGGUGACUCtt-3′
PTEN siRNA	5′-GGCGUAUACAGGAACAAUAtt-3′	5′-CAAGATGATGTTTGAAACTAtt-3′
Negative Control siRNA	5′-UUCUCCGAACGUGUCACGUtt-3′	5′-ACGUGACACGUUCGGAGAAtt-3′
**Plasmid for target genes**	**Sequence-sense**	**Sequence-anti-sense**
homo-HIF1A	5′-CGAGCTCAAGCTTCGAATTCTATGGAGGGCGCCGGCGGCGCGAAC-3′	5′-TATCTAGATCCGGTGGATCCCTAGTTAACTTGATCCAAAGCTCTG-3′
homo-SP1	5′-ACGGGCCCTCTAGACTCGAGATGAGCGACCAAGATCACTCC-3′	5′-AGTCACTTAAGCTTGGTACCGAGAAGCCATTGCCACTGATATTAATG-3′
homo-H19	5′-ACGGGCCCTCTAGACTCGAGGGGAGGGGGTGGGATG-3′	5′-AGTCCAGTGTGGTGGAATTCTTGCTGTAACAGTGTTTATTGATG-3′
