# Supplementary material for: Hypoxia induces H19 expression through direct and indirect Hif-1α activity, promoting oncogenic effects in glioblastoma
Source: Sci Rep. 2017 Mar 22;7:45029. doi: 10.1038/srep45029 (PMC5361208; doi:10.1038/srep45029)
Supplement: Supplementary Figures [file srep45029-s1.docx]

**Hypoxia induces H19 expression through direct and indirect Hif-1α activity, promoting oncogenic effects in glioblastoma**

Weining Wu^1^*, Qi Hu^1^*, Er Nie^1^*, Tianfu, Yu^1^, Youzhi Wu^1^, Tongle Zhi^1^, Kuan Jiang^1^, Feng Shen^1^, Yingyi Wang^1^, Junxia Zhang^1^, Yongping You^1^

^1^ Department of Neurosurgery, The First Affiliated Hospital of Nanjing Medical University, Nanjing, Jiangsu 210000, China.

^*^ These authors contributed equally to this work.

Corresponding author: Yongping You, Professor, Chief physician of Neurosurgery Department, The First Affiliated Hospital of Nanjing Medical University, Nanjing, China.

Correspondence: YYPL9@njmu.edu.cn.

**
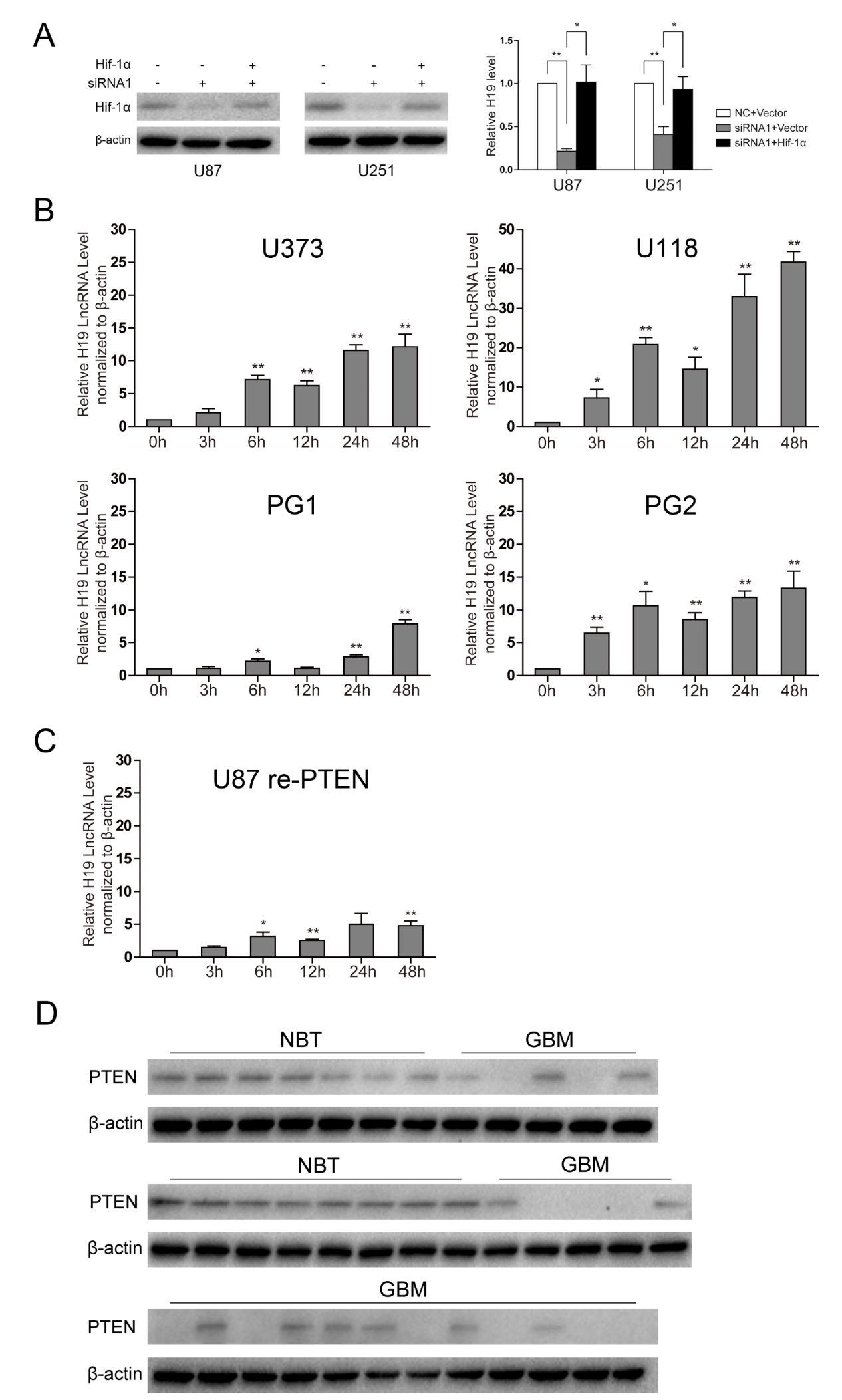
**

**Supplementary Figure 1.**

**A.** Re-express Hif-1α in U87 and U251 cells transfected with Hif-1α siRNA1 under hypoxia for 24h. All experiments were repeated three times with similar results. (*p<0.05, **p<0.01)

**B.** The different phenotypes of H19 induction under hypoxia in multiple GBM cell lines including U373, U118 and two primary GBM cell lines from two patients. All experiments were repeated three times with similar results. (*p<0.05, **p<0.01)

**C.** The phenotype of hypoxia-stimulated H19 induction in U87 cells transfected with PTEN plasmid. All experiments were repeated three times with similar results. (*p<0.05, **p<0.01)

**D.** The PTEN status in human clinical specimens were analyzed by western blot. The expression of PTEN was normalized to β-actin.

**
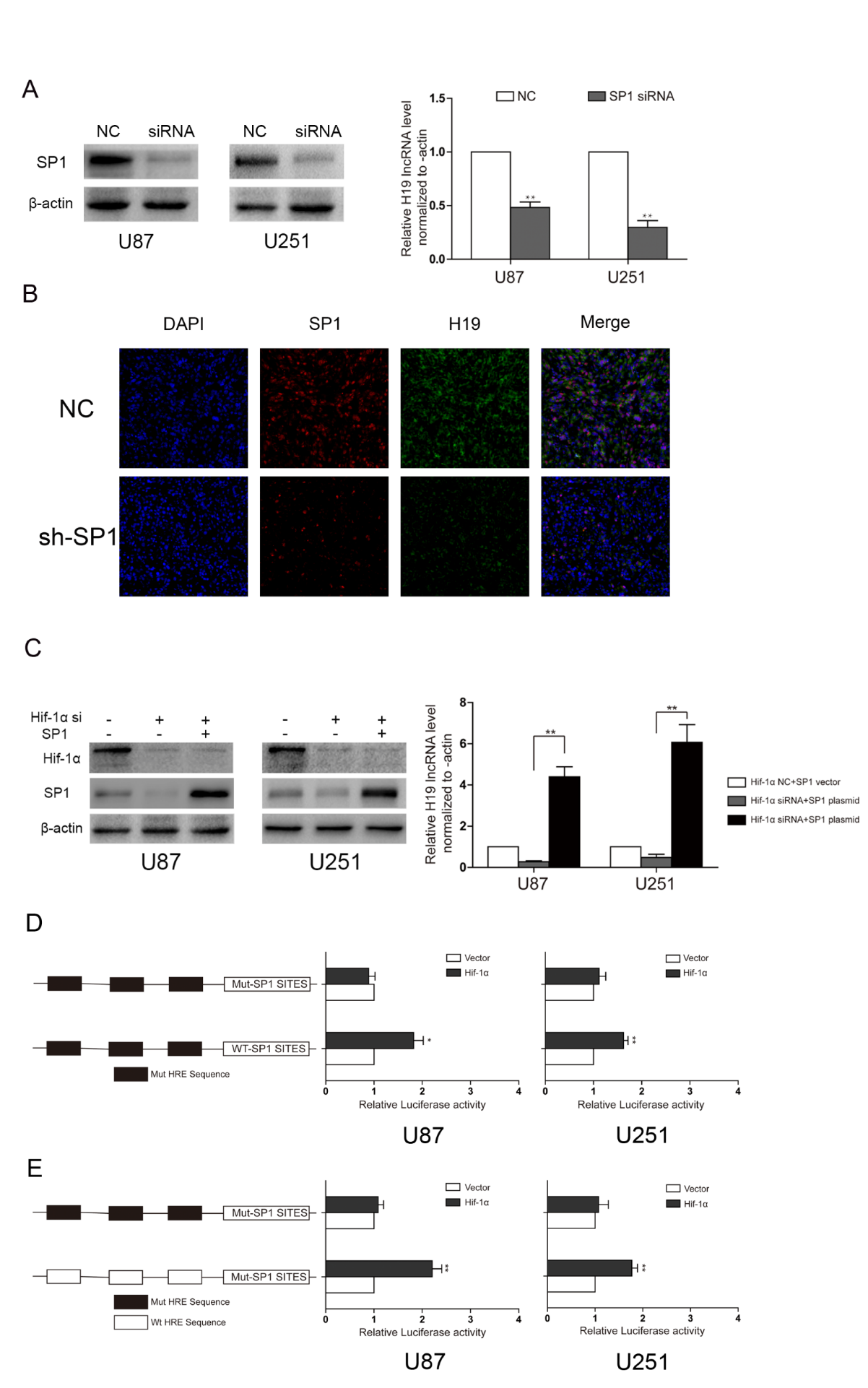
**

**Supplementary Figure 2.**

**A** U87 and U251 cells transfected with a negative control siRNA or SP1 siRNA before culture under hypoxia for 24h;

**B** U87 cells transfected with a negative control or sh-SP1 lentivirus were injected subcutaneously into nude mice. The frozen sections of xenografts from nude mice were analyzed by double-stained IF.

**C** U87 and U251 cells co-transfected with Hif-1α siRNA and the SP1 expression plasmid with hypoxic treatment for 24h. All experiments were repeated three times with similar results. (*p<0.05, **p<0.01)

**D.** Two reporter plasmids harboring wild type or mutant GC-boxes and mutated HREs were co-transfected with the Hif-1α expression plasmid or empty vector and the Renilla plasmid into U87 and U251 cells. Relative luciferase activity was analyzed after 24h treatment. (*p<0.05, **p<0.01)

**E.** Two reporter plasmids harboring wild type or mutant HREs and mutated SP1 binding sites were co-transfected with the Hif-1α expression plasmid or empty vector and the Renilla plasmid into U87 and U251 cells. Relative luciferase activity was analyzed after 24h treatment. (*p<0.05, **p<0.01)


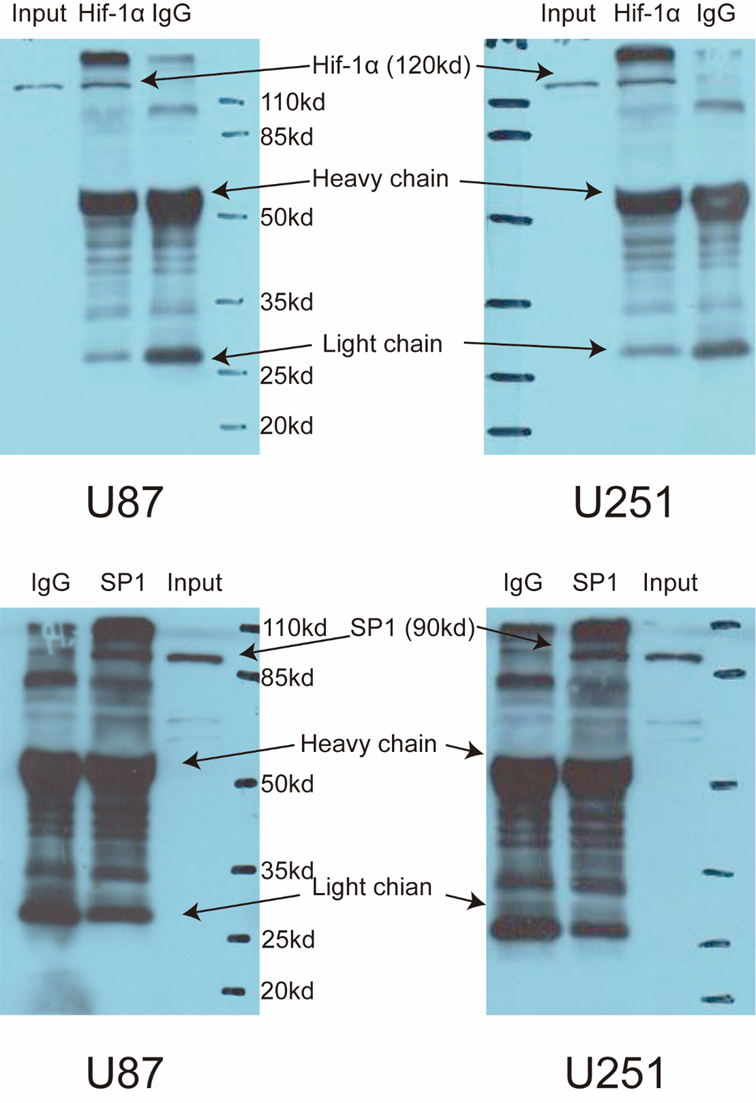


**Supplementary CHIP assays validation**

Chromatin complexes in Input, IgG and relevant gene (Hif-1α or SP1) group were examined by western blot assays with respective antibody. It is confirmed that

Hif-1α or SP1 successfully immunoprecipitate chromatin complexes before QPCR assays in U87 and U251 cells.
